# Maggots in External Ear

**Published:** 1874-05

**Authors:** J. A. Meek

**Affiliations:** Jonesboro, Arkansas


### MAGGOTS IN THE EXTERNAL EAR.

*Editor Atlanta Medical Journal:*

In a last year's Journal I noticed from Prof. A. W. Calhoun what I regarded at the time as a very novel case. He reported maggots found in the meatus auditorius externus. At the time I read this report I had never heard of anything of the kind before, and remember of wondering how it could be. Since reading the case of Dr. Calhoun, however, or at least the one reported by him, I have heard of a case analogus, occurring in the neighborhood of this place. The subject was a small son of a Mr. Hearn, who resides three miles from Jonesboro. He was plowing in the field when an insect came buzzing along and entered the ear. He did not know at the time what it was, but subsequently learned it was a blue-bottle-fly. He finally succeeded in removing it, but not until it had deposited its eggs. The maggots hatched in due time. The pain was intolerable. The boy had a high fever and was very restless. Upon an examination nine large active maggots were found lodged in the meatus externus. They were promptly removed by the father of the boy, when the pain and fever immediately subsided and the boy made a rapid recovery.

Yours, Respectfully,

J. A. MEEK.

*Jonesboro, Arkansas, February*, 1874.

